# To Dr. Bradley

**Published:** 1800-07

**Authors:** Charles Cooke

**Affiliations:** Glocester


### To Dr. BRADLEY.

SIR,

IF I had waited for the present increafing numbers of impartial teftimonies and indubitable facts in favour of Vaccine Inoculation, I need not have troubled you to infert this letter in your ufeful Publication; but as, in the onfet, I did (with more zeal than prudence) oppofe, what I then confidered an innovation in practice, (by confounding the uncertain effects of the cafual, and, perhaps, fpurious cow-pox, with the real advantages evidently arifing from this difeafe when inoculated) I now think it right, in juftice to Dr. Jenner and the medical public, to declare,

That, in the courfe of my practice, I had occafion to make trial, and do approve of vaccine inoculation; yet, I think it fhould only be conducted by practitioners who have taken proper care to afcertain the genuine difeafe.

I remain, with great refpect,

SIR,

Your obliged and conftant reader,

